# Association of Prenatal Exposure to Ambient and Traffic-Related Air Pollution With Newborn Thyroid Function: Findings From the Children’s Health Study

**DOI:** 10.1001/jamanetworkopen.2018.2172

**Published:** 2018-09-14

**Authors:** Caitlin G. Howe, Sandrah P. Eckel, Rima Habre, Mariam S. Girguis, Lu Gao, Frederick W. Lurmann, Frank D. Gilliland, Carrie V. Breton

**Affiliations:** 1 Department of Preventive Medicine, Keck School of Medicine, University of Southern California, Los Angeles; 2 Sonoma Technology, Petaluma, California

## Abstract

**Importance:**

Thyroid hormones are critical for fetal growth and development. Prenatal particulate matter (PM) air pollution exposure has been associated with altered newborn thyroid function, but other air pollutants have not been evaluated, and critical windows of exposure are unknown.

**Objectives:**

To investigate the association of prenatal exposure to ambient and traffic-related air pollutants with newborn thyroid function and identify critical windows of exposure.

**Design, Setting, and Participants:**

This cohort study used data from 2050 participants in the Children’s Health Study. Statistical analyses were conducted from 2017 to 2018 using pregnancy and birth data from 1994 to 1997 for a subset of participants recruited from schools in 13 southern California communities in 2002 to 2003 when participants were 5 to 7 years of age. Participants were included in statistical analyses if they could be linked to their newborn blood spot and had complete monthly exposure measures for at least 1 air pollutant across pregnancy.

**Exposures:**

Prenatal monthly averages of ambient (PM diameter <2.5 μm [PM_2.5_] or <10 μm [PM_10_], nitrogen dioxide, and ozone) and traffic-related (freeway, nonfreeway, and total nitrogen oxides) air pollutant exposures were determined using inverse distance-squared weighting of central monitoring data and the California Line Source Dispersion model, respectively.

**Main Outcomes and Measures:**

Newborn heel-stick blood spot total thyroxine (TT4) measures were acquired retrospectively from the California Department of Public Health.

**Results:**

Participants included 2050 newborns (50.5% male), with a median (interquartile range) age of 20 (15-29) hours. The majority of newborns were Hispanic white (1202 [58.6%]) or non-Hispanic white (638 [31.1%]). Sixty-six (3.2%) were black and 144 (7.0%) were from other racial/ethnic groups. The mean (SD) newborn TT4 measure was 16.2 (4.3) μg/dL. A 2-SD increase in prenatal PM_2.5_ (16.3 μg/m^3^) and PM_10_ (22.2 μg/m^3^) was associated with a 1.2-μg/dL (95% CI, 0.5-1.8 μg/dL) and 1.5-μg/dL (95% CI, 0.9-2.1 μg/dL) higher TT4 measure, respectively, in covariate-adjusted linear regression models. Other pollutants were not consistently associated with newborn TT4. Distributed lag models revealed that PM_2.5_ exposure during months 3 to 7 of pregnancy and PM_10_ exposure during months 1 to 8 of pregnancy were associated with significantly higher newborn TT4 concentrations (*P* < .05).

**Conclusions and Relevance:**

Prenatal PM exposure, particularly in early pregnancy and midpregnancy, is associated with higher newborn TT4 concentrations. Future studies should assess the health implications of PM-associated differences in newborn TT4 concentrations.

## Key Points

**Question:**

Are prenatal ambient and traffic-related air pollutant exposures associated with newborn total thyroxine concentrations, and are there critical windows of exposure?

**Findings:**

In a cohort study of a subset of 2050 newborns from the Children’s Health Study in southern California, an increase of 2 standard deviations in prenatal exposure to particulate matter in air pollution was associated with higher newborn total thyroxine measures. Months 3 to 7 and 1 to 8 of pregnancy were identified as critical windows of exposure to particulate matter and associated higher thyroxine levels.

**Meaning:**

The fetal thyroid gland may be susceptible to particulate matter air pollution toxicity, especially during early pregnancy and midpregnancy.

## Teaser

This cohort study examines the association between prenatal exposure to ambient and traffic-related air pollution and newborn total thyroxine concentrations and investigates critical gestational windows of exposure during pregnancies.

## Introduction

Thyroid hormones are critical for regulating fetal growth and metabolism and play important roles in neurodevelopment.^1^ Even subtle changes in maternal thyroid function during pregnancy have been associated with reduced fetal growth and cognitive deficits in children, with detrimental effects observed for both low and excess levels of thyroid hormones.^2,3,4,5,6,7,8,9,10^

Two major hormones are secreted from the thyroid gland: thyroxine (T4), the predominant circulating form of thyroid hormone, and triiodothyronine (T3), the metabolically active form of thyroid hormone, which is largely derived from T4.^11^ While the majority of circulating thyroid hormone (>99%) is bound to plasma proteins, only the free fraction is biologically active.^12^ The release of T4 and T3 from the thyroid gland is controlled by thyroid stimulating hormone (TSH), which in turn is secreted by the pituitary gland and tightly regulated by circulating levels of T4 and T3 via a negative feedback loop.^11^ Because fetal thyroid gland development largely occurs during the first trimester,^1^ the fetus is highly dependent on maternal T4 during this period.^13^ However, the fetus begins producing its own supply of T4 at the end of the first trimester,^14^ with production increasing until approximately 35 to 37 weeks of pregnancy.^1^

Several classes of environmental toxicants, including dioxins, polychlorinated biphenyls, certain pesticides, and heavy metals (eg, cadmium), have been associated with altered newborn thyroid function.^15,16,17^ Prenatal tobacco smoke exposure, an important source of indoor particulate matter (PM) pollution, has also been associated with altered newborn thyroid measures, especially lower cord blood TSH concentrations, with some evidence of associations with T3 and T4.^18,19^ It has therefore been hypothesized that ambient PM air pollution may similarly alter newborn thyroid function.^20^ In support of this, a recent study conducted in the Environmental Influence on Early Aging (ENVIRONAGE) birth cohort observed significant associations between prenatal exposure to PM with diameter less than 2.5 μm (PM_2.5_), assessed at the third trimester, and cord blood thyroid hormone concentrations.^20^ However, relationships between other air pollutants and newborn thyroid function were not investigated, and the most vulnerable windows of pregnancy have not been identified. The objectives of the current study were to (1) examine whether ambient and traffic-related air pollution exposures during pregnancy are associated with altered newborn thyroid function, assessed using newborn heel-stick blood spot total T4 (TT4) measures, which include both the free and protein-bound fractions of T4, and (2) identify sensitive windows of exposure in the Children’s Health Study (CHS) from southern California.

## Methods

### Participants

The CHS consists of a series of southern California–based cohorts that have been described previously.^21,22^ For the current study, we selected all CHS participants enrolled at 5 to 7 years of age between 2002 and 2003 from public schools in 13 communities across southern California who could be matched to a California birth record and had complete information for at least 1 air pollutant across pregnancy (n = 2410). Newborn heel-stick blood spot TT4 concentrations were requested retrospectively from the California Department of Public Health (CDPH) for participants. However, 15 participants could not be matched by the CDPH to a newborn blood spot and 1 participant was born after December 1997, when the CDPH switched to using TSH measures for routine congenital hypothyroidism screening. Therefore, TT4 concentrations were obtained for a total of 2394 participants. Of these participants, 2050 were included in primary statistical analyses because they were singleton births and had plausible gestational age at birth values, including a 2-week buffer for late-term newborns (147-322 gestational days), and complete information for all other relevant covariates. This study was reviewed and approved by the institutional review board at the University of Southern California. Informed consent (from parents) and assent (from children) were obtained for all CHS participants. The study followed the Strengthening the Reporting of Observational Studies in Epidemiology (STROBE) reporting guideline for cohort studies.

### Prenatal Air Pollution Exposures

Prenatal air pollutant exposures were estimated for CHS participants on a monthly basis based on their geocoded residence, which was obtained using birth certificate and residential history questionnaire data. Residential addresses were geocoded using a combination of the ESRI geocoding database and software (ESRI Inc), Google Earth, and the Texas A&M geocoder.^23^ Ambient air quality monitoring data from the US Environmental Protection Agency’s Air Quality System^24^ and from the CHS^25^ were used for this study. Federal Reference Method continuous monitors were used to measure nitrogen dioxide and ozone and Federal Reference Method or Federal Equivalent Method monitors were used to measure PM_2.5_ and PM with diameter less than 10 μm (PM_10_). Monthly averages of pollutants were calculated from the daily data if available for 75% or more of the month. Participants’ monthly air pollutant measures were then spatially interpolated, using inverse distance-squared weighting, as described previously.^26^ Monthly averages of traffic-related nitrogen oxide concentrations for freeway and nonfreeway roads were determined with the California Line Source Dispersion model,^27^ using EMFAC2011 vehicle emission rates, traffic volume, road geometry, and meteorological conditions, including wind speed and direction, pollution mixing heights, and atmospheric stability.^27^ Of the 2050 participants, 274 (13.4%) reported moving during the pregnancy period or during the birth month, which was accounted for in exposure assessment. Air pollutant pregnancy averages were calculated based on the participant’s gestational age at birth and when in the month he or she was born (described in more detail in eMethods in the Supplement). Monthly and pregnancy averages of air pollutants were standardized to 2 SDs of their means prior to inclusion in statistical models.

### Total T4 Measures

Newborn heel-stick blood spot TT4 concentrations were measured by the CDPH using the Micromedic Concept 4 automated radioimmunological test (ICN Biomedical Inc). The CDPH also provided the newborn’s age (in hours) at blood spot collection and the time (in days) from blood spot collection until TT4 measurement.

### Participant Characteristics

Information on maternal parity, birth weight, gestational age at birth, mode of delivery, date of birth, precipitous (<3 hours) or prolonged (>20 hours) labors, principal payment source for prenatal care, and pregnancy complications (including preeclampsia, pregnancy-induced hypertension, eclampsia, chronic hypertension, renal disease, pyelonephritis, anemia, cardiac disease, acute or chronic lung disease, diabetes, Rh sensitization, hemoglobinopathy, uterine bleeding before labor, polyhydramnios or oligohydramnios, incompetent cervix, premature labor, genital herpes, other sexually transmitted diseases, hepatitis B, and rubella) was obtained from participants’ birth records. Because southern California is characterized by 2 seasons, warm (born on or after March 16 and before October 1) and cool (born on or after October 1 and before March 16), season of birth was classified into 1 of these categories. Information on maternal tobacco smoke use during pregnancy (ever vs never), paternal tobacco smoke use during pregnancy (ever vs never), maternal education, newborn’s sex, and total household income category were obtained by questionnaire. Information on the child’s race and ethnicity was also acquired by questionnaire. These variables were anticipated to be important confounders for the original aims of the CHS, which focused on respiratory health. The child’s race (white, black, Asian, Hawaiian or Pacific Islander, North American Indian, other, or mixed) and ethnicity (Hispanic or non-Hispanic) were reported by his or her parents. These categories were defined by CHS principal investigators. For the current study, the race and ethnicity variables were combined and categories were collapsed to create 1 variable with the following categories: non-Hispanic white, Hispanic white, black, and other.

### Temperature Data

Daily average temperature data were acquired for participant birth years (1995-1997) from the US Environmental Protection Agency Air Quality Data Mart^24^ for all of southern California. Monthly averages were calculated if at least 21 days of data were available. Spatial interpolation was conducted using inverse distance-squared weighting using a minimum of 12 stations and a maximum distance of 50 km. Average monthly temperature at birth was assigned to participants based on their geocoded address.

### Statistical Analysis

Statistical analyses were conducted between 2017 and 2018, using pregnancy and birth data from 1994 to 1997 for a subset of 2050 CHS participants. Participants were recruited between 2002 and 2003 at 5 to 7 years of age from schools across 13 southern California communities.

Statistical analyses were conducted using R statistical software version 3.5.0 (R Project for Statistical Computing). Spearman correlations were used to evaluate relationships between prenatal air pollutant exposures. Associations between pregnancy averages of pollutants and newborn TT4 concentrations were evaluated in linear regression models, with 1 model per pollutant. Assumptions of linear regression models, including homoscedasticity and normal distributions of the outcome and model residuals, were examined. Because there was some evidence of heteroscedasticity, robust linear regression models were also run and results were compared with ordinary least-squares regression results. To ascertain the independent effects of ambient pollutants, 2-pollutant and 3-pollutant models were evaluated; given the high correlation between PM_2.5_ and PM_10_ (eFigure 1 in the Supplement), these pollutants were not included in the same models. Variance inflation factors were determined for each pollutant to identify potential collinearity in multipollutant models. Potential interactions were evaluated between each pollutant and newborn’s sex, birth weight, gestational age at birth, maternal tobacco smoke exposure, and season of birth using cross-product terms.

Vulnerable windows of exposure were formally investigated for air pollutants that remained significantly associated with TT4 in 3-pollutant models. Distributed lag models (DLMs), which account for both current and past values of the exposure, were used to examine monthly averages of air pollutants in relation to TT4. These models were conducted using the dlnm package in R.^28^ These analyses were restricted to full- and late-term newborns (n = 1880), such that air pollutant exposures during the first 9 months of pregnancy could be evaluated. For primary analyses, natural cubic spline DLMs with 3 *df* (ie, 1 knot placed at the median lag period: month 5) were evaluated. The results were then compared with models that instead placed a knot at month 3 of pregnancy, as this is when the fetal thyroid gland begins producing T4^14^ and was therefore hypothesized to mark the beginning of a potentially vulnerable window of exposure. In sensitivity analyses, natural cubic spline DLMs with 4 *df* (ie, 2 equal-spaced knots) were also examined to capture any additional complexity in the exposure-lag-response relationship. The DLMs were compared using the Akaike information criterion (AIC).

All models were adjusted for hypothesized confounders and predictors of newborn thyroid function, including maternal age, parity, education (categorized as completed high school vs did not complete high school), maternal smoking status during pregnancy, newborn sex, season of birth (warm vs cool season), race/ethnicity, gestational age at birth, age at blood spot collection, and community at enrollment (represented by 12 dummy variables). In sensitivity analyses, models examining pregnancy averages of each air pollutant were also adjusted for newborn’s birth month (instead of season of birth), average temperature during the birth month, the mode of delivery (vaginal vs cesarean), reported total household income category at the time of enrollment (which included a category for 252 participants who did not know their household income or who declined to report it), the principal payment source used for prenatal care (categorized as none/government, health insurance, and other), paternal smoking status during pregnancy, and time from blood spot collection until TT4 analysis. Additional models were also run excluding the following: (1) preterm pregnancies, (2) cesarean deliveries, (3) newborns exposed in utero to tobacco smoke, (4) participants with pregnancy complications, (5) participants with precipitous (<3 hours) or prolonged (>20 hours) labors, (6) communities with fewer than 100 participants (Alpine and Santa Barbara), (7) participants whose blood spots were collected more than 48 hours after birth, and (8) participants who moved to a new residence during the pregnancy period or birth month.

Two-sided *P* values less than .05 were considered statistically significant for all models. *P* values from cross-product terms were adjusted for multiple testing based on the false discovery rate.

## Results

### Study Participant Characteristics

Characteristics of study participants are shown in the Table. The majority of newborns were Hispanic white (1202 [58.6%]) or non-Hispanic white (638 [31.1%]). Sixty-six (3.2%) were black and 144 (7.0%) were from other racial/ethnic groups. There were relatively equal proportions of male (50.5%) and female (49.5%) newborns. The median (interquartile range) age at blood spot collection was 20 (15-29) hours after birth, and the mean (SD) time between blood spot collection and TT4 analysis was 3 (2) days. The mean (SD) TT4 measure was 16.2 (4.3) μg/dL (to convert to nanomoles per liter, multiply by 12.871), similar to what has been observed in other populations.^2,29^ Newborn TT4 concentrations were also positively and significantly correlated with birth weight (ρ = 0.06; *P* = .007), which has also been observed previously.^29^ A total of 170 newborns (8.3%) were preterm (characteristics shown in eTable 1 in the Supplement). The subset of full- and late-term newborns included in DLM analyses was very similar to the larger study population (Table). Distributions of air pollutant exposures were similar across pregnancy (eFigures 2-8 in the Supplement).

**Table.** Descriptive Statistics of Study Participants

Exposures and Characteristics	Full Study Sample (n = 2050)	Full-term and Late-term (n = 1880)
**Prenatal Air Pollutant Exposures**		
Particulate matter, μg/m^3^		
<2.5 μm^a^		
Mean (SD)	21.5 (8.2)	21.5 (8.2)
Median (IQR)	24.2 (14.8-27.5)	24.3 (14.8-27.5)
<10 μm		
Mean (SD)	42.0 (11.1)	42.1 (11.0)
Median (IQR)	41.8 (37.3-48.0)	41.9 (37.4-48.0)
Nitrogen dioxide, ppb		
Mean (SD)	30.0 (10.8)	30.0 (10.8)
Median (IQR)	33.0 (22.8-37.4)	33.0 (22.9-37.4)
Ozone, ppb		
Mean (SD)	44.9 (12.0)	45.0 (11.9)
Median (IQR)	44.3 (34.0-54.2)	44.6 (34.1-54.3)
Nitrogen oxides, ppb^b^		
Total		
Mean (SD)	27.2 (23.5)	27.1 (23.0)
Median (IQR)	21.1 (11.6-36.3)	21.0 (11.7-36.1)
Freeway		
Mean (SD)	17.1 (20.3)	16.9 (19.7)
Median (IQR)	11.2 (4.2-22.6)	11.1 (4.3-22.4)
Nonfreeway		
Mean (SD)	10.2 (7.3)	10.2 (7.3)
Median (IQR)	8.6 (5.2-13.8)	8.6 (5.2-13.7)
**Newborn and Pregnancy Characteristics**		
Age at blood spot collection, h		
Mean (SD)	27 (35)	26 (35)
Median (IQR)	20 (15-29)	20 (15-28)
Time from blood spot collection until TT4 measurement, d		
Mean (SD)	3 (2)	3 (2)
Median (IQR)	3 (2-4)	3 (2-4)
Heel stick TT4, μg/dL		
Mean (SD)	16.2 (4.3)	16.4 (4.2)
Median (IQR)	15.9 (13.3-18.8)	16.0 (13.4-19.0)
Gestational age, d		
Mean (SD)	277 (15)	280 (10)
Median (IQR)	278 (270-285)	279 (273-286)
Sex, No. (%)		
Male	1036 (50.5)	950 (50.5)
Female	1014 (49.5)	930 (49.5)
Race/ethnicity, No. (%)		
Hispanic white	1202 (58.6)	1089 (57.9)
Non-Hispanic white	638 (31.1)	599 (31.9)
Black	66 (3.2)	59 (3.1)
Other	144 (7.0)	133 (7.1)
Season of birth, No. (%)		
Warm	1151 (56.1)	1050 (55.9)
Cool	899 (43.9)	830 (44.1)
Mode of delivery, No. (%)		
Vaginal	1628 (79.4)	1491 (79.3)
Cesarean	422 (20.6)	389 (20.7)
Pregnancy complication, No. (%)^c^		
Yes	195 (9.5)	163 (8.7)
No	1855 (90.5)	1717 (91.3)
Labor duration, No. (%)		
Precipitous (<3 h)	9 (0.4)	9 (0.5)
Normal (3-20 h)	2030 (99.0)	1860 (98.9)
Prolonged (>20 h)	11 (0.5)	11 (0.6)
Principal payment source for prenatal care, No. (%)		
None or government	754 (36.8)	671 (35.7)
Health insurance	1233 (60.1)	1153 (61.3)
Other	63 (3.1)	56 (3.0)
**Maternal and Family Characteristics**		
Age, mean (SD), y	27 (6)	27 (6)
Education, No. (%)		
Completed at least high school	1486 (72.5)	1382 (73.5)
Did not complete high school	564 (27.5)	498 (26.5)
Maternal smoking status during pregnancy, No. (%)		
Ever	145 (7.1)	128 (6.8)
Never	1905 (92.9)	1752 (93.2)
Paternal smoking status during pregnancy, No. (%)		
Ever	307 (15.0)	278 (14.8)
Never	1685 (82.2)	1548 (82.3)
Did not report	58 (2.8)	54 (2.9)
Parity, No. (%)		
Parous	1306 (63.7)	1206 (64.1)
Nulliparous	744 (36.3)	674 (35.9)
Total household income, No. (%)		
<$7500	91 (4.4)	83 (4.4)
$7500-$14 999	157 (7.7)	144 (7.7)
$15 000-$29 999	304 (14.8)	271 (14.4)
$30 000-$49 999	359 (17.5)	333 (17.7)
$50 000-$74 999	340 (16.6)	319 (17.0)
$75 000-$99 999	268 (13.1)	254 (13.5)
≥$100 000	279 (13.6)	261 (13.9)
Do not know or did not report	252 (12.3)	215 (11.4)

Abbreviations: IQR, interquartile range; TT4, total thyroxine.

SI conversion factor: To convert TT4 to nanomoles per liter, multiply by 12.871.

^a^ Sample sizes were 2046 for full sample and 1876 for full- and late-term subset.

^b^ Sample sizes were 1989 for full sample and 1824 for full- and late-term subset.

^c^ Preeclampsia or pregnancy-induced hypertension, eclampsia, chronic hypertension, renal disease, pyelonephritis, anemia, cardiac disease, acute or chronic lung disease, diabetes, Rh sensitization, hemoglobinopathy, uterine bleeding before labor, polyhydramnios or oligohydramnios, incompetent cervix, premature labor, genital herpes, other sexually transmitted diseases, hepatitis B, or rubella.

### Individual Models for Pregnancy Averages of Air Pollutants

**Figure 1.**
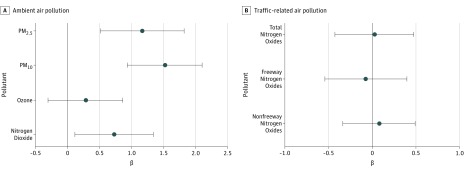
Individual Models for Pregnancy Averages of Air Pollutants β coefficients and 95% confidence intervals are shown for associations between pregnancy averages of ambient (A) and traffic-related (B) air pollution exposures and newborn blood spot total thyroxine concentrations. Pollutants were evaluated in individual models using linear regression. All models were adjusted for newborn’s sex, newborn’s race/ethnicity, gestational age at birth, season of birth, maternal parity, maternal age, maternal education, maternal tobacco smoke use during pregnancy, age at newborn blood spot collection, and the community of the participant at recruitment. The β coefficient represents the difference in total thyroxine (micrograms per deciliter) for a 2-SD difference in the pollutant. Analysis included 2050 participants for particulate matter with a diameter less than 10 μm (PM_10_), nitrogen dioxide, and ozone; 2046 participants for particulate matter with a diameter less than 2.5 μm (PM_2.5_); and 1989 participants for total, freeway, and nonfreeway nitrogen oxides.

In individual pollutant models, after adjusting for hypothesized confounders, a 2-SD difference in exposure to PM_2.5_ (equivalent to 16.3 μg/m^3^), PM_10_ (equivalent to 22.2 μg/m^3^), and nitrogen dioxide (equivalent to 21.6 parts per billion) during pregnancy was associated with a difference in newborn TT4 of 1.2 μg/dL (95% CI, 0.5-1.8 μg/dL; *P* < .001), 1.5 μg/dL (95% CI, 0.9-2.1 μg/dL; *P* < .001), and 0.7 μg/dL (95% CI. 0.1-1.3 μg/dL; *P* = .02), respectively (Figure 1). In contrast, prenatal ozone and traffic-related air pollution exposures were not significantly associated with TT4 concentrations (Figure 1). Results from robust linear regression models were similar (eTable 2 in the Supplement).

Associations between PM_2.5_ or PM_10_ exposure and newborn TT4 concentration remained statistically significant with similar magnitudes of association in sensitivity analyses (eTable 3 in the Supplement).

No significant interactions were observed between any of the pollutants and maternal smoking, newborn’s sex, birth weight, gestational age at birth, or season of birth (false discovery rate–adjusted *P* ≥ .86 for all cross-product terms evaluated).

### Multipollutant Models for Pregnancy Averages of Ambient Pollutants

**Figure 2.**
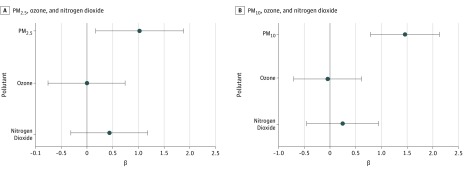
Three-Pollutant Models for Pregnancy Averages of Air Pollutants β coefficients and 95% confidence intervals are shown for associations between pregnancy averages of ambient pollutants and newborn blood spot total thyroxine concentrations from 3-pollutant models. A, Particulate matter with a diameter less than 2.5 μm (PM_2.5_), nitrogen dioxide, and ozone were included simultaneously in 1 linear regression model. B, Particulate matter with a diameter less than 10 μm (PM_10_), nitrogen dioxide, and ozone were included simultaneously in 1 linear regression model. All models were adjusted for newborn’s sex, newborn’s race/ethnicity, gestational age at birth, season of birth, maternal parity, maternal age, maternal education, maternal tobacco smoke use during pregnancy, age at newborn blood spot collection, and the community of the participant at recruitment. The β coefficient represents the difference in total thyroxine (micrograms per deciliter) for a 2-SD difference in the pollutant. Analysis included 2050 participants for model A and 2046 participants for model B.

While there was some evidence of collinearity in 3-pollutant models, particularly for models including PM_2.5_ (eTable 4 in the Supplement), variance inflation factors were smaller for 2-pollutant models, which yielded similar results (Figure 2; eTable 5 in the Supplement). Results from multipollutant models were similar to those from individual pollutant models (Figure 1 and Figure 2; eTable 5 in the Supplement), except that prenatal nitrogen dioxide exposure was no longer significantly associated with newborn TT4 concentration after adjusting for either PM_2.5_ or PM_10_.

### Distributed Lag Models for Monthly PM Exposures

**Figure 3.**
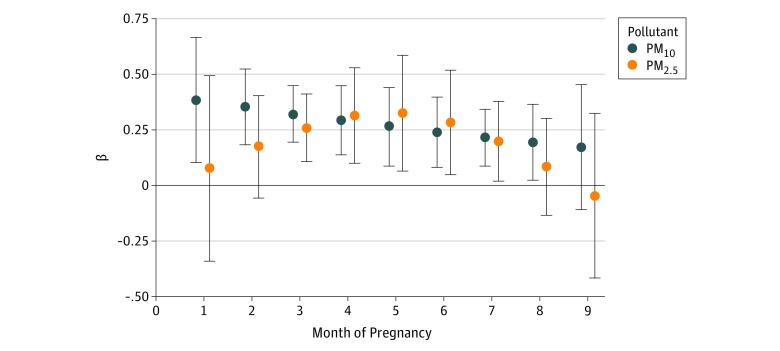
Distributed Lag Model Results for PM_2.5_ and PM_10_ β coefficients and 95% confidence intervals from distributed lag models are shown for associations between particulate matter with a diameter less than 10 μm (PM_10_) or less than 2.5 μm (PM_2.5_) at each month of pregnancy and total thyroxine concentrations at birth, adjusted for newborn’s sex, newborn’s race/ethnicity, gestational age at birth, season of birth, maternal parity, maternal age, maternal education, maternal tobacco smoke use during pregnancy, age at newborn blood spot collection, and the community of the participant at recruitment, among full-term and late-term newborns. Individual distributed lag models were used to evaluate PM_2.5_ and PM_10_, using a natural cubic spline function with 3 *df* (1 knot at month 5 of pregnancy). β coefficients represent the difference in newborn total thyroxine (micrograms per deciliter) for a 2-SD difference in the pollutant. Analysis included 1880 participants for the PM_10_ model and 1876 participants for the PM_2.5_ model.

Results of DLMs for PM_2.5_ and PM_10_ are shown in Figure 3 and are shown for other pollutants in eFigures 9 to 13 in the Supplement. Exposure to PM_2.5_ during months 3 to 7 of pregnancy was positively and significantly associated with newborn TT4 concentrations, while PM_10_ exposure was positively and significantly associated with newborn TT4 concentrations between months 1 and 8 of pregnancy (*P* < .05 for all). The strongest association between PM_2.5_ and TT4 was observed for exposure at month 5 of pregnancy (β, 0.3; 95% CI, 0.1-0.6), and the strongest association between PM_10_ and TT4 was observed for exposure at month 1 of pregnancy (β, 0.4; 95% CI, 0.1-0.7). Results were similar when a knot was placed at the third month of pregnancy (PM_2.5_ AIC = 10 667.7 and PM_10_ AIC = 10 674.2) rather than at the median lag period (month 5) (PM_2.5_ AIC = 10 667.7 and PM_10_ AIC = 10 674.2) and when 2 equal-spaced knots were evaluated (PM_2.5_ AIC = 10 669.6 and PM_10_ AIC = 10 676.1) (eFigures 14 and 15 in the Supplement).

## Discussion

In the current study, prenatal exposure to ambient PM_2.5_ and PM_10_ air pollution was associated with higher newborn TT4 concentration. However, traffic-related air pollution and ozone were not significantly associated with newborn TT4 concentration, and nitrogen dioxide did not remain significantly associated with newborn TT4 concentration after adjusting for PM_2.5_ or PM_10_. Distributed lag models revealed that the positive association between PM_2.5_ and newborn TT4 concentration emerged at the end of the first trimester. In contrast, PM_10_ exposure throughout most of pregnancy (months 1-8) was associated with higher TT4 concentrations.

Several previous studies have observed that tobacco smoke, an important source of indoor PM air pollution, alters both adult and fetal thyroid function, with the majority of studies observing inverse relationships between tobacco smoke exposure and TSH, with some evidence also of higher thyroid hormone levels among smokers and smoke-exposed newborns.^18,19,30^ In the current study, prenatal ambient PM air pollution exposure was associated with higher newborn TT4 measures. To our knowledge, only 1 previous smaller study (n = 499), conducted by Janssen et al^20^ in the ENVIRONAGE birth cohort, investigated associations between ambient PM air pollution exposure and newborn thyroid function. Their study focused on third trimester PM_2.5_ exposure and observed an inverse association with TSH, a positive association with free T3 concentrations in cord blood,^20^ and inverse relationships between PM_2.5_ and both free T4 and the ratio of free T4 to free T3 in cord blood. The authors therefore hypothesized that PM_2.5_ may increase the conversion of T4 to T3 by inducing type 2 deiodinase activity.^20^ While interesting, this hypothesis could not be examined in the current study because the CDPH does not measure T3 as part of their routine screening for congenital hypothyroidism. Although Janssen et al^20^ observed an inverse association between PM_2.5_ and free T4, while in contrast PM_2.5_ was positively associated with TT4 in the current study, these findings are not necessarily inconsistent. Because the majority of T4 is bound to serum transport proteins, such as thyroxin-binding globulin (TBG), free T4 makes up only a small fraction (0.02%) of TT4.^12^ While we are unaware of any studies that have investigated the association of air pollutants with thyroxine-binding proteins, tobacco smoke use has been associated with higher TBG levels among adult men.^31^ Therefore, it is possible that PM increases newborn TBG concentrations, which in turn could lead to higher TT4 but lower free T4 measures in newborns. Given that thyroid hormone levels are very tightly regulated through a negative feedback loop, it is unclear whether these transient effects would be captured over long periods of time. However, studies of prenatal air pollution and newborn thyroid function may benefit from measuring TBG in addition to free and total thyroid hormone concentrations.

While Janssen et al observed significant associations between third trimester PM_2.5_ and cord blood thyroid measures, they did not additionally examine or adjust for PM_2.5_ exposure at previous points in pregnancy. In the current study, we used DLMs, which account for both current and past exposures, and observed that PM_10_ exposure throughout most of pregnancy was associated with higher newborn TT4 measures, whereas the association between PM_2.5_ and newborn TT4 was isolated to the midpregnancy period. Because the fetus begins producing its own supply of T4 in midpregnancy, this may be a particularly vulnerable period for the developing thyroid gland.^14^ This may also be a window of sensitivity for the maternal thyroid gland, as maternal thyroid function changes dramatically during pregnancy, with both T4 and TBG levels increasing in early pregnancy and plateauing in midpregnancy.^32^ It is therefore possible that alterations in maternal thyroid function may partially or fully explain the associations observed between PM exposure and higher TT4 concentrations in newborns. For example, given that T4 can only cross the placenta when it is protein bound, PM-induced increases in maternal TBG could increase placental transfer of T4 to the fetus, thereby elevating newborn TT4 concentrations. Although PM_2.5_ exposure in late pregnancy was not found to be associated with maternal thyroid hormone concentrations in the ENVIRONAGE cohort,^20^ to our knowledge the association of early-pregnancy or midpregnancy PM exposure with maternal thyroid function has not been examined.

Discrepancies in the vulnerable windows identified for PM_10_ vs PM_2.5_ may be driven by differences in particle size, which affects deposition patterns in the airways, as well as particle composition. For example, in southern California certain crustal materials and trace elements are more abundant in coarse PM, whereas metals and other toxicants derived from vehicular emissions tend to be more abundant in PM_2.5_,^33,34^ which could affect distinct aspects of thyroid gland development and function.

### Limitations and Strengths

Importantly, there were several limitations of the current study. First, only TT4 measures were available for most study participants. Therefore, other indicators of thyroid function and related parameters (eg, TBG) could not be examined in relation to prenatal air pollution, which limits the ability to interpret the observed associations between prenatal PM exposure and newborn TT4 concentrations. Additionally, while models were adjusted for a large number of potential confounders, including several indicators of social and economic status, the possibility of residual confounding cannot be ruled out. Furthermore, because information on maternal thyroid function was not available for the current study, women with overt hyperthyroidism and hypothyroidism could not be excluded from analyses, and potential mediation of prenatal PM associations with newborn TT4 concentrations by maternal thyroid function could not be evaluated. Because the current study population was predominantly Hispanic white and non-Hispanic white and resided in southern California, results from the current study may not be generalizable to other populations.

The current study also had unique strengths. The CHS was designed to capture a wide range of multiple air pollutant exposures throughout southern California, many of which had not, to our knowledge, been examined previously for association with newborn thyroid function. Because air pollution exposure histories were assembled for CHS participants, including during the prenatal period, exposure to multiple pollutants could be examined across pregnancy in relation to newborn TT4 concentrations, which allowed for the identification of potentially susceptible windows of exposure. Another major strength of the study was the large sample size (n = 2050), which yielded sufficient statistical power to detect relatively small, but highly significant, PM-associated differences in newborn TT4 concentrations after accounting for a large number of potential confounders and precision variables.

While the clinical significance of the PM-associated differences in newborn TT4 concentrations observed in this study (1.2-μg/dL and 1.5-μg/dL higher TT4 concentrations for a 2-SD increase in PM_2.5_ or PM_10_, respectively) is currently unknown, even small differences in maternal thyroid function during pregnancy have been associated with reduced fetal growth and adverse effects on neurodevelopment.^7,8^ Although most studies have focused on thyroid hormone deficiencies during pregnancy, several studies have observed an inverse U-shaped relationship between maternal thyroid hormone levels during pregnancy and offspring outcomes, such as birth weight and cognition.^3,4,5,6^ There is also some evidence that higher newborn T4 concentrations adversely affect cognitive outcomes. For example, higher newborn heel-stick blood spot TT4 concentrations have been associated with lower visual recognition memory scores at 6 months of age,^2^ and higher postnatal free T4 concentrations have been associated with worse neurodevelopmental outcomes at 7 years of age.^35^ Therefore, excess levels of thyroid hormones in early life may also be detrimental. Nevertheless, additional studies will be needed to assess the potential health consequences of PM-associated differences in newborn blood spot TT4 concentrations.

## Conclusions

Findings from the current study suggest that prenatal PM exposure is associated with higher newborn TT4 concentrations. Although PM_10_ exposure throughout most of pregnancy was associated with higher newborn TT4 concentrations, PM_2.5_ exposure in midpregnancy only was associated with higher newborn TT4 concentrations. Prenatal nitrogen dioxide, ozone, and traffic-related air pollution exposures were not consistently associated with newborn TT4 concentrations.

## Notes

Supplement. **eMethods.** Additional Information on Prenatal Air Pollution Exposure Assignments. **eTable 1.** Characteristics of Preterm Newborns (n = 170) **eTable 2.** Comparison of Ordinary Least Squares and Robust Linear Regression Results **eTable 3.** Sensitivity Analyses for Associations between Prenatal Particulate Matter Exposures and Newborn TT4 **eTable 4.** Variance Inflation Factors for Three-Pollutant Models **eTable 5.** Two-Pollutant Models for Associations between Pregnancy Averages of Air Pollutants and Newborn Total Thyroxine **eFigure 1.** Spearman Correlations between Pregnancy Averages of Air Pollutants. **eFigure 2.** Box and Whisker Plots of Particulate Matter <2.5 μm (PM_2.5_) Across Pregnancy **eFigure 3.** Box and Whisker Plots of Particulate Matter <10 μm (PM_10_) Across Pregnancy **eFigure 4.** Box and Whisker Plots of Nitrogen Dioxide (NO_2_) Across Pregnancy **eFigure 5.** Box and Whisker Plots of Ozone (O_3_) Across Pregnancy **eFigure 6.** Box and Whisker Plots of Total Nitrogen Oxides (NO_x_) Across Pregnancy **eFigure 7.** Box and Whisker Plots of Freeway Nitrogen Oxides (NO_x_) Across Pregnancy **eFigure 8.** Box and Whisker Plots of Non-Freeway Nitrogen Oxides (NO_x_) Across Pregnancy **eFigure 9.** Distributed Lag Model Results for Nitrogen Dioxide (NO_2_) **eFigure 10.** Distributed Lag Model Results for Ozone (O_3_) **eFigure 11.** Distributed Lag Model Results for Total Nitrogen Oxides (NO_x_) **eFigure 12.** Distributed Lag Model Results for Freeway Nitrogen Oxides (NO_x_) **eFigure 13.** Distributed Lag Model Results for Non-Freeway Nitrogen Oxides (NO_x_) **eFigure 14.** Sensitivity Analyses for Particulate Matter <2.5 μm (PM_2.5_) Distributed Lag Models **eFigure 15.** Sensitivity Analyses for Particulate Matter <10 μm (PM_10_) Distributed Lag Models
