# Supplementary material for: Association of Prenatal Exposure to Ambient and Traffic-Related Air Pollution With Newborn Thyroid Function: Findings From the Children’s Health Study
Source: JAMA Netw Open. 2018 Sep 14;1(5):e182172. doi: 10.1001/jamanetworkopen.2018.2172 (PMC6324507; doi:10.1001/jamanetworkopen.2018.2172)
Supplement: Supplement. — eMethods. Additional Information on Prenatal Air Pollution Exposure Assignments. eTable 1. Characteristics of Preterm Newborns (n = 170) eTable 2. Comparison of Ordinary Least Squares and Robust Linear Regression Results eTable 3. Sensitivity Analyses for Associations between Prenatal Particulate Matter Exposures and Newborn TT4 eTable 4. Variance Inflation Factors for Three-Pollutant Models eTable 5. Two-Pollutant Models for Associations between Pregnancy Averages of Air Pollutants and Newborn Total Thyroxine eFigure 1. Spearman Correlations between Pregnancy Averages of Air Pollutants. eFigure 2. Box and Whisker Plots of Particulate Matter <2.5 μm (PM2.5) Across Pregnancy eFigure 3. Box and Whisker Plots of Particulate Matter <10 μm (PM10) Across Pregnancy eFigure 4. Box and Whisker Plots of Nitrogen Dioxide (NO2) Across Pregnancy eFigure 5. Box and Whisker Plots of Ozone (O3) Across Pregnancy eFigure 6. Box and Whisker Plots of Total Nitrogen Oxides (NOx) Across Pregnancy eFigure 7. Box and Whisker Plots of Freeway Nitrogen Oxides (NOx) Across Pregnancy eFigure 8. Box and Whisker Plots of Non-Freeway Nitrogen Oxides (NOx) Across Pregnancy eFigure 9. Distributed Lag Model Results for Nitrogen Dioxide (NO2) eFigure 10. Distributed Lag Model Results for Ozone (O3) eFigure 11. Distributed Lag Model Results for Total Nitrogen Oxides (NOx) eFigure 12. Distributed Lag Model Results for Freeway Nitrogen Oxides (NOx) eFigure 13. Distributed Lag Model Results for Non-Freeway Nitrogen Oxides (NOx) eFigure 14. Sensitivity Analyses for Particulate Matter <2.5 μm (PM2.5) Distributed Lag Models eFigure 15. Sensitivity Analyses for Particulate Matter <10 μm (PM10) Distributed Lag Models [file jamanetwopen-1-e182172-s001.pdf]

Supplementary Online Content

Howe CG, Eckel SP, Habre R, et al. Association of Prenatal Exposure to Ambient and Traffic-Related Air Pollution With Newborn Thyroid Function: Findings From the Children's Health Study. *JAMA Netw Open.* 2018;1(5):e182172. doi:10.1001/jamanetworkopen.2018.2172

eMethods. Additional Information on Prenatal Air Pollution Exposure Assignments.

eTable 1. Characteristics of Preterm Newborns (n = 170)

eTable 2. Comparison of Ordinary Least Squares and Robust Linear Regression Results

eTable 3. Sensitivity Analyses for Associations between Prenatal Particulate Matter Exposures and Newborn TT4

eTable 4. Variance Inflation Factors for Three-Pollutant Models

eTable 5. Two-Pollutant Models for Associations between Pregnancy Averages of Air Pollutants and Newborn Total Thyroxine

eFigure 1. Spearman Correlations between Pregnancy Averages of Air Pollutants.

eFigure 2. Box and Whisker Plots of Particulate Matter <2.5 μm (PM2.5) Across Pregnancy

eFigure 3. Box and Whisker Plots of Particulate Matter <10 μm (PM10) Across Pregnancy

eFigure 4. Box and Whisker Plots of Nitrogen Dioxide (NO2) Across Pregnancy

eFigure 5. Box and Whisker Plots of Ozone (O3) Across Pregnancy

eFigure 6. Box and Whisker Plots of Total Nitrogen Oxides (NOx) Across Pregnancy

eFigure 7. Box and Whisker Plots of Freeway Nitrogen Oxides (NOx) Across Pregnancy

eFigure 8. Box and Whisker Plots of Non-Freeway Nitrogen Oxides (NOx) Across Pregnancy

eFigure 9. Distributed Lag Model Results for Nitrogen Dioxide (NO2)

eFigure 10. Distributed Lag Model Results for Ozone (O3)

eFigure 11. Distributed Lag Model Results for Total Nitrogen Oxides (NOx)

eFigure 12. Distributed Lag Model Results for Freeway Nitrogen Oxides (NOx)

eFigure 13. Distributed Lag Model Results for Non-Freeway Nitrogen Oxides (NOx)

© 2018 Howe CG et al. *JAMA Network Open.*

eFigure 14. Sensitivity Analyses for Particulate Matter <2.5 μm (PM2.5) Distributed Lag Models

eFigure 15. Sensitivity Analyses for Particulate Matter <10 μm (PM10) Distributed Lag Models

This supplementary material has been provided by the authors to give readers additional information about their work.

© 2018 Howe CG et al. *JAMA Network Open.*

eMethods. Additional Information on Prenatal Air Pollution Exposure Assignments

To account for the fact that some newborns were born early in a month, and others late in a month, participants were classified into two categories: those born before the 15th of a month versus those born on or after the 15th of a month. To account for differences in gestational age at birth, participants were also classified into five gestational age categories: (six months: 5.5 ≤ gestational age < 6.5, seven months: 6.5 ≤ gestational age < 7.5, eight months: 7.5 ≤ gestational age < 8.5, nine months: 8.5 ≤ gestational age < 9.5, ten months: 9.5 ≤ gestational age < 10.5). For babies born early in a month, air pollutant pregnancy averages were calculated by taking the average of monthly air pollutant measures for each month that the participant was *in utero* prior to his/her birth month (e.g. for a baby born early in a month, with a gestational age at birth of eight months, monthly air pollutant measures for the eight months prior to the birth month were averaged). For babies born late in a month, prenatal air pollutant averages were calculated by taking the average of monthly air pollutant measures for the participant's birth month and each month prior to that that he/she was *in utero* (e.g. for a baby born late in a month, with a gestational age at birth of eight months, the air pollutant measure for the participant's birth month, and measures for the seven months prior to that, were averaged).

© 2018 Howe CG et al. *JAMA Network Open.*

eTable 1. Characteristics of Preterm Newborns (n = 170)

**Prenatal Air Pollutant Exposures**	**Mean (SD) or No. (%)**	**Median (IQR)**
Particulate Matter < 2.5 μm, μg/m^3[a]	21.1 (8.2)	23.0 (14.5-27.7)
Particulate Matter < 10 μm, μg/m^3	41.5 (11.7)	41.1 (37.0-48.1)
Nitrogen Dioxide, parts per billion (ppb)	29.2 (11.1)	31.2 (21.7-36.5)
Ozone, ppb	44.3 (13.1)	42.1 (33.4-53.2)
Total Nitrogen Oxides (NO_x), ppb[b]	29.0 (28.9)	22.7 (10.7-40.4)
Freeway NO_x, ppb[b]	18.9 (25.8)	11.4 (3.3-24.8)
Non-Freeway NO_x, ppb[b]	10.0 (7.4)	8.2 (4.7-14.6)
**Newborn and Pregnancy Characteristics**		
Age at blood spot collection, hours	38 (38)	24 (17-41)
Time from blood spot collection until TT4 analysis, days	3 (2)	3 (2-4)
Heel stick total thyroxine (TT4), μg/dl	14.6 (4.9)	14.5 (11.5-17.2)
Gestational age, days	244 (15)	250 (239-254)
Sex		
Male	86 (50.6)	
Female	84 (49.4)	
Race/Ethnicity		
Hispanic white	113 (66.5)	
Non-Hispanic white	39 (22.9)	
Black	7 (4.1)	
Other	11 (6.5)	
Season of birth		
Warm	101 (59.4)	
Cool	69 (40.6)	
Mode of Delivery		
Vaginal	137 (80.6)	
Caesarean section	33 (19.4)	
Pregnancy Complication[d]		
Yes	32 (18.8)	
No	138 (81.2)	
Labor Duration		
Precipitous (<3 hours)	0 (0.0)	
Normal (3-20 hours)	170 (100.0)	

© 2018 Howe CG et al. *JAMA Network Open.*

Prenatal Air Pollutant Exposures	Mean (SD) or No. (%)	Median (IQR)
Prolonged (>20 hours)	0 (0.0)	
Principal Payment Source for Prenatal Care[e]		
None or government	83 (48.8)	
Health insurance	80 (47.1)	
Other	7 (4.1)	
**Maternal and Family Characteristics**		
Age, years	26 (6)	26 (20-30)
Education		
Completed at least high school	104 (61.2)	
Did not complete high school	66 (38.8)	
Maternal Smoking Status During Pregnancy		
Ever smoked during pregnancy	17 (10.0)	
Never smoked during pregnancy	153 (90.0)	
Paternal Smoking Status During Pregnancy		
Ever smoked during the pregnancy	29 (17.1)	
Never smoked during the pregnancy	137 (80.6)	
Did not report	4 (2.4)	
Parity		
Parous	100 (58.8)	
Nulliparous	70 (41.2)	
Total Household Income		
<$7,500	8 (4.7)	
$7,500-$14,999	13 (7.6)	
$15,000-$29,999	33 (19.4)	
$30,000-$49,999	26 (15.3)	
$50,000-$74,999	21 (12.4)	
$75,000-$99,999	14 (8.2)	
≥$100,000	18 (10.6)	
Don’t Know or Did Not Report	37 (21.8)	

© 2018 Howe CG et al. *JAMA Network Open.*

eTable 2. Comparison of Ordinary Least Squares and Robust Linear Regression Results[a]

Pollutant	Ordinary Least Squares Regression		Robust Linear Regression	
	β (95% CI)	P	β (95% CI)	P
Particulate Matter < 2.5 μm, n = 2046	1.2 (0.5, 1.8)	$4x10^{-4}$	1.2 (0.5, 1.8)	$8x10^{-4}$
Particulate Matter < 10 μm, n = 2050	1.5 (0.9, 2.1)	$2x10^{-7}$	1.5 (1.0, 2.1)	$7x10^{-8}$
Nitrogen Dioxide, n = 2050	0.7 (0.1, 1.3)	0.02	0.5 (-0.08, 1.1)	0.07
Ozone, n = 2050	0.3 (-0.3, 0.9)	0.34	0.3 (-0.3, 0.8)	0.36
Total Nitrogen Oxides (NO_x), n = 1989	0.0 (-0.4, 0.5)	0.91	-0.1 (-0.5, 0.4)	0.77
Freeway NO_x, n = 1989	0.0 (-0.4, 0.5)	0.82	-0.1 (-0.5, 0.3)	0.79
Non-Freeway NO_x, n = 1989	-0.1 (-0.5, 0.4)	0.76	0.0 (-0.5, 0.4)	0.88

[a]Associations between pregnancy averages of air pollutants and newborn total thyroxine (TT4) concentration, adjusting for baby's sex, baby's race/ethnicity, gestational age at birth, season of birth, maternal parity, maternal age, maternal education, maternal tobacco smoke use during pregnancy, age at newborn blood spot collection, and the community of the participant at recruitment. Effect estimates represent differences in newborn TT4 (μg/dL) for a 2 SD difference in the pollutant.

© 2018 Howe CG et al. *JAMA Network Open.*

eTable 3. Sensitivity Analyses for Associations[a] between Prenatal Particulate Matter Exposures and Newborn TT4

Model	$PM_{2.5}$			PM_{10}		
	n	β (95% CI)	P	n	β (95% CI)	P
Primary Analysis	2046	1.2 (0.5, 1.8)	3.7×10^{-4}	2050	1.5 (0.9, 2.1)	2.3×10^{-7}
Restricted to Full-/Late-Term Pregnancies	1876	1.5 (0.8, 2.1)	2.0×10^{-5}	1880	1.7 (1.1, 2.3)	3.2×10^{-8}
Restricted to Vaginal Births	1624	1.0 (0.3, 1.8)	6.0×10^{-3}	1628	1.5 (0.9, 2.2)	3.8×10^{-6}
Excluding Newborns Exposed to Tobacco Smoke *In Utero*	1901	1.3 (0.6, 2.0)	1.3×10^{-4}	1905	1.6 (1.0, 2.2)	1.1×10^{-7}
Excluding Pregnancies with Complications[b]	1852	1.3 (0.6, 1.9)	1.7×10^{-4}	1855	1.7 (1.1, 2.3)	6.3×10^{-8}
Excluding Pregnancies with Precipitous or Prolonged Labors[c]	2027	1.2 (0.5, 1.8)	4.6×10^{-4}	2030	1.5 (0.9, 2.1)	3.7×10^{-7}
Excluding Participants Who Moved During Pregnancy	1776	1.5 (0.8, 2.2)	2.5×10^{-5}	1776	1.7 (1.0, 2.3)	9.5×10^{-8}
Excluding Participants Residing in Communities with <100 Participants (Alpine and Santa Barbara)	2027	1.2 (0.6, 1.8)	2.9×10^{-4}	2031	1.5 (1.0, 2.1)	1.8×10^{-7}
Excluding Participants Whose Bloodspots Were Collected >48 Hours Post-Birth	1894	1.2 (0.5, 1.8)	6.0×10^{-4}	1897	1.4 (0.8, 2.4)	2.7×10^{-6}
Adjusting for Month, Rather than Season, of Birth	2046	1.0 (0.4, 1.7)	.002	2050	1.5 (0.9, 2.1)	3.0×10^{-7}
Additionally Adjusting for Temperature at Birth	2046	1.1 (0.4, 1.7)	.001	2050	1.5 (0.9, 2.1)	2.1×10^{-7}
Additionally Adjusting for Mode of Delivery	2046	1.1 (0.5, 1.8)	5.2×10^{-4}	2050	1.5 (0.9, 2.1)	3.3×10^{-7}
Additionally Adjusting for Household Income	2046	1.2 (0.5, 1.8)	4.3×10^{-4}	2050	1.5 (0.9, 2.1)	3.4×10^{-7}

© 2018 Howe CG et al. *JAMA Network Open.*

Model	$PM_{2.5}$			PM_{10}		
	n	β (95% CI)	P	n	β (95% CI)	P
Additionally Adjusting for Paternal Smoking Status During Pregnancy	2046	1.2 (0.5, 1.8)	3.5×10^{-4}	2050	1.5 (0.9, 2.1)	2.2×10^{-7}
Additionally Adjusting for Prenatal Care Principal Payment Source[d]	2046	1.2 (0.5, 1.8)	3.9×10^{-4}	2050	1.5 (0.9, 2.1)	2.7×10^{-7}
Additionally Adjusting for Time From Blood Spot Collection Until TT4 Measurement	2046	1.2 (0.5, 1.8)	4.5×10^{-4}	2050	1.5 (0.9, 2.1)	2.7×10^{-7}

[a]Results are from linear regression models, adjusted for baby's sex, baby's race/ethnicity, gestational age at birth, season of birth (unless otherwise specified), maternal parity, maternal age, maternal education, maternal tobacco smoke use during pregnancy, age at newborn blood spot collection, and the community of the participant at recruitment. Effect estimates represent differences in newborn total thyroxine (TT4) (μg/dL) for a 2 SD difference in the pollutant.

[b]Pre-eclampsia/pregnancy-induced hypertension, eclampsia, chronic hypertension, renal disease, pyelonephritis, anemia, cardiac disease, acute or chronic lung disease, diabetes, Rh sensitization, hemoglobinopathy, uterine bleeding before labor, polyhydramnios/oligohydramnios, incompetent cervix, premature labor, genital herpes, other sexually transmitted diseases, hepatitis B, or rubella.

[c]Precipitous labor defined as <3 hours. Prolonged labor defined as >20 hours.

[d]Principal payment source for prenatal care was categorized into one of three categories: (health insurance, none/government, other).

© 2018 Howe CG et al. *JAMA Network Open.*

eTable 4. Variance Inflation Factors for Three-Pollutant Models[a]

	Variance Inflation Factor
**$PM_{2.5}$, NO_2, O_3 Model (n = 2046)**	
$PM_{2.5}$	4.1
NO_2	5.4
O_3	4.2
**PM_{10}, NO_2, O_3 Model (n = 2050)**	
PM_{10}	3.3
NO_2	3.6
O_3	3.2

[a]Results are from linear regression models, including pregnancy averages of particulate matter < 2.5 μm ($PM_{2.5}$), nitrogen dioxide (NO_2), and ozone (O_3) or particulate matter < 10 μm (PM_{10}), NO_2, and O_3 in one regression model. Models were adjusted for baby's sex, baby's race/ethnicity, gestational age at birth, season of birth (unless otherwise specified), maternal parity, maternal age, maternal education, maternal tobacco smoke use during pregnancy, age at newborn blood spot collection, and the community of the participant at recruitment. Effect estimates represent differences in newborn total thyroxine (TT4) (μg/dL) for a 2 SD difference in the pollutant.

© 2018 Howe CG et al. *JAMA Network Open.*

eTable 5. Two-Pollutant Models for Associations[a] Between Pregnancy Averages of Air Pollutants and Newborn Total Thyroxine

	β (95% CI)	P	Variance Inflation Factor
**Particulate Matter < 2.5 μm ($PM_{2.5}$) and Nitrogen Dioxide (NO_2) Model (n = 2046)**			
$PM_{2.5}$	1.0 (0.3, 1.7)	$3x10^{-3}$	3.5
NO_2	0.4 (-0.2, 1.1)	.18	3.1
**$PM_{2.5}$ and Ozone (O_3) Model (n = 2046)**			
$PM_{2.5}$	1.3 (0.6, 2.0)	$5x10^{-3}$	3.9
O_3	-0.2 (-0.9, 0.4)	.49	3.1
**Particulate Matter < 10 μm (PM_{10}) and NO_2 Model (n = 2050)**			
PM_{10}	1.4 (0.8, 2.0)	$3x10^{-6}$	2.8
NO_2	0.3 (-0.4, 0.9)	.41	3.0
**PM_{10} and O_3 Model (n = 2050)**			
PM_{10}	1.6 (1.0, 2.2)	$3x10^{-7}$	2.7
O_3	-0.1 (-0.7, 0.5)	.65	2.7
**NO_2 and O_3 Model (n = 2050)**			
NO_2	0.9 (0.3, 1.5)	$6x10^{-3}$	3.0
O_3	0.5 (-0.1, 1.1)	.09	2.7

[a]Results are from linear regression models, adjusted for baby's sex, baby's race/ethnicity, gestational age at birth, season of birth (unless otherwise specified), maternal parity, maternal age, maternal education, maternal tobacco smoke use during pregnancy, age at newborn blood spot collection, and the community of the participant at recruitment. Effect estimates represent differences in newborn total thyroxine (μg/dL) for a 2 standard deviation difference in the pollutant.

© 2018 Howe CG et al. *JAMA Network Open.*

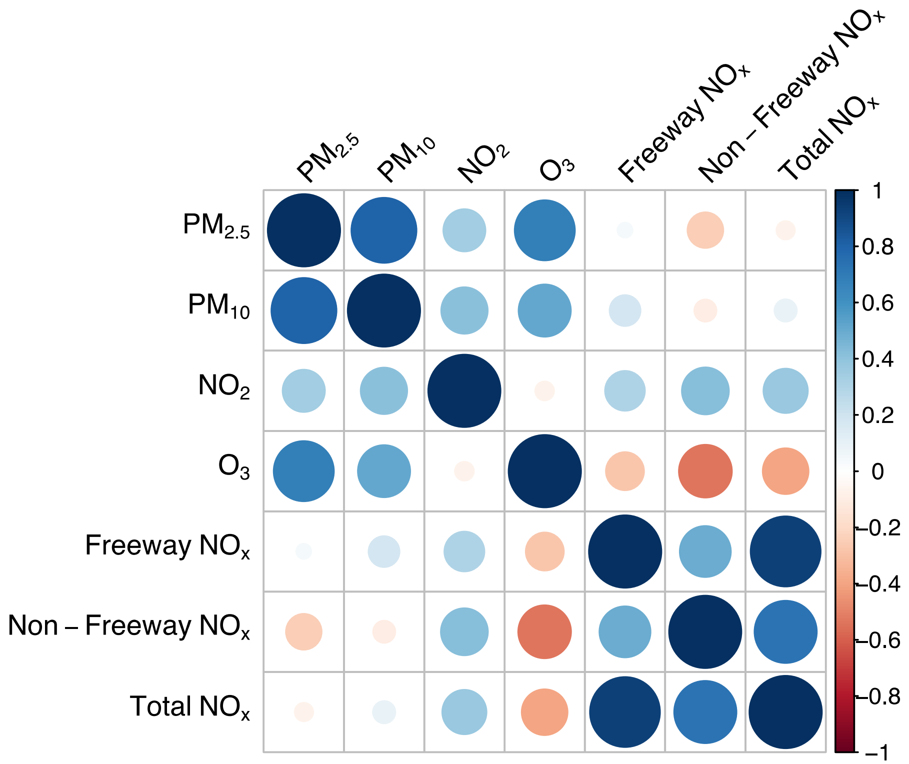

eFigure 1. Spearman Correlations Between Pregnancy Averages of Air Pollutants. Darker shades represent stronger correlations. Blue shades indicate positive correlations, while red shades indicate negative correlations, as outlined in corresponding key. n = 1989. Abbreviations: NO_2, Nitrogen Dioxide; NO_x, Nitrogen Oxides; O_3, Ozone; $PM_{2.5}$, Particulate Matter < 2.5 µm; PM_{10}, Particulate Matter < 10 µm;

© 2018 Howe CG et al. *JAMA Network Open.*

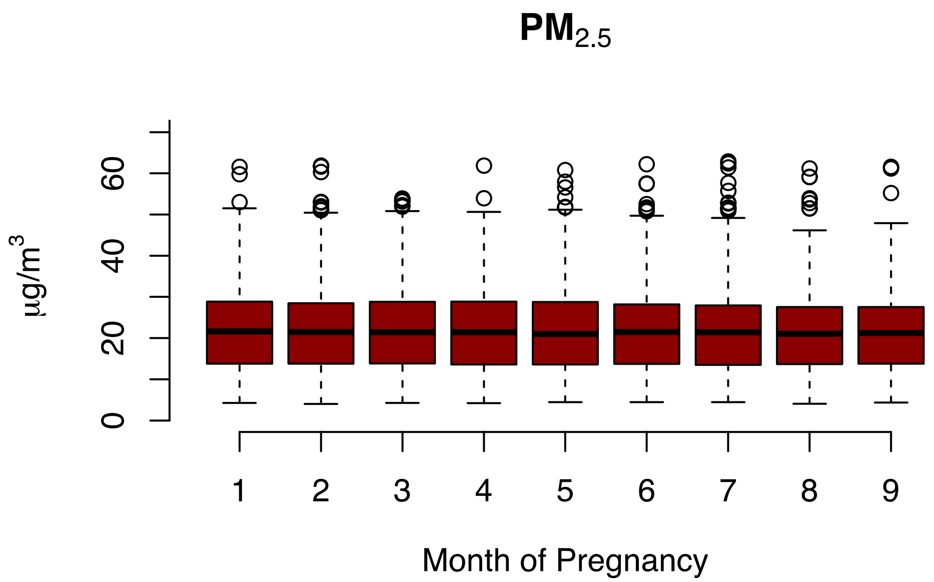

eFigure 2. Box and Whisker Plots of Particulate Matter < 2.5 μm ($PM_{2.5}$) Across Pregnancy. Box and whisker plots showing the distribution of $PM_{2.5}$ (μg/m^3) at each of the first nine months of pregnancy. The boxes represent the interquartile ranges for $PM_{2.5}$. The horizontal black line dividing the box represents the median. Lower whiskers represent the first quartile $PM_{2.5}$ value - 1.5 x the interquartile range for $PM_{2.5}$ at that month. Upper whiskers represent the third quartile $PM_{2.5}$ value + 1.5 x the interquartile range for $PM_{2.5}$ at that month. Open circles represent $PM_{2.5}$ values that are greater than upper whisker $PM_{2.5}$ values.

© 2018 Howe CG et al. *JAMA Network Open.*

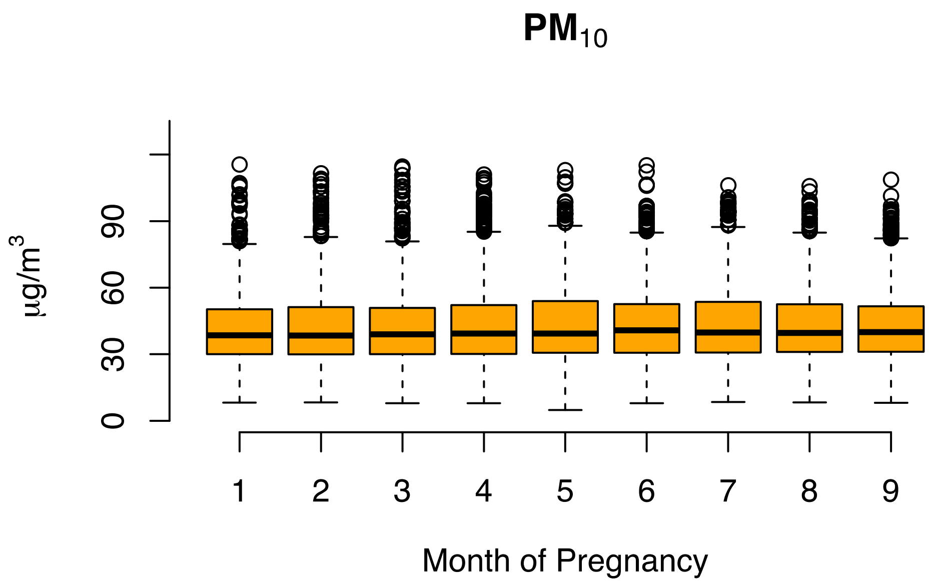

eFigure 3. Box and Whisker Plots of Particulate Matter < 10 μm (PM_{10}) Across Pregnancy. Box and whisker plots showing the distribution of PM_{10} (μg/m^3) at each of the first nine months of pregnancy. The boxes represent the interquartile ranges for PM_{10}. The horizontal black line dividing the box represents the median. Lower whiskers represent the first quartile PM_{10} value - 1.5 x the interquartile range for PM_{10} at that month. Upper whiskers represent the third quartile PM_{10} value + 1.5 x the interquartile range for PM_{10} at that month. Open circles represent PM_{10} values that are greater than upper whisker PM_{10} values.

© 2018 Howe CG et al. *JAMA Network Open.*

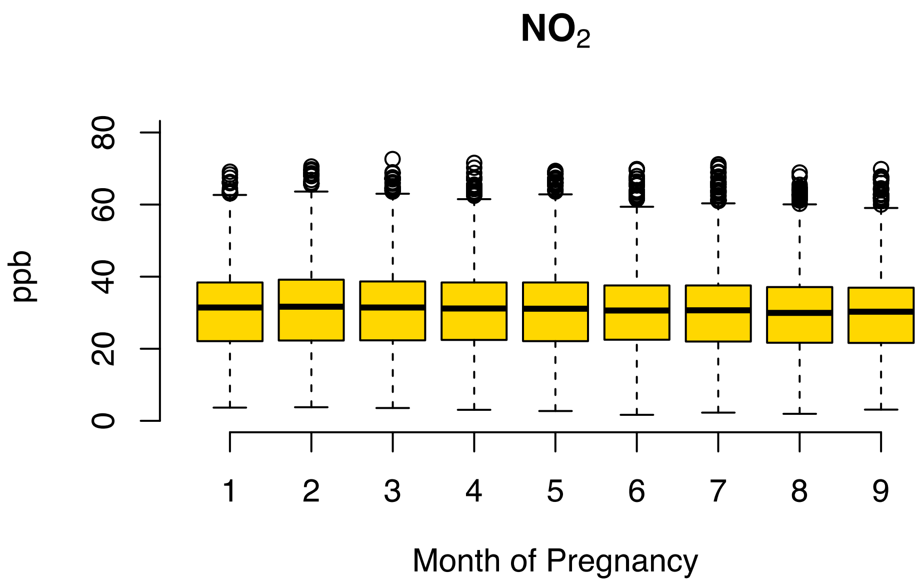

eFigure 4. Box and Whisker Plots of Nitrogen Dioxide (NO_2) Across Pregnancy. Box and whisker plots showing the distribution of NO_2 (parts per billion (ppb)) at each of the first nine months of pregnancy. The boxes represent the interquartile ranges for NO_2. The horizontal black line dividing the box represents the median. Lower whiskers represent the first quartile NO_2 value - 1.5 x the interquartile range for NO_2 at that month. Upper whiskers represent the third quartile NO_2 value + 1.5 x the interquartile range for NO_2 at that month. Open circles represent NO_2 values that are greater than upper whisker NO_2 values. Abbreviations: ppb, parts per billion.

© 2018 Howe CG et al. *JAMA Network Open.*

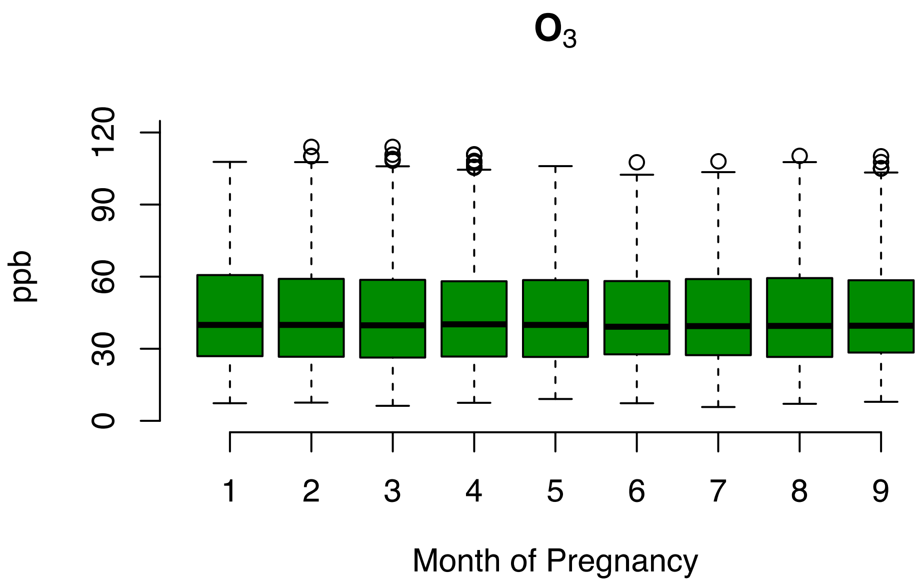

eFigure 5. Box and Whisker Plots of Ozone (O_3) Across Pregnancy. Box and whisker plots showing the distribution of O_3 (parts per billion (ppb)) at each of the first nine months of pregnancy. The boxes represent the interquartile ranges for O_3. The horizontal black line dividing the box represents the median. Lower whiskers represent the first quartile O_3 value - 1.5 x the interquartile range for O_3 at that month. Upper whiskers represent the third quartile O_3 value + 1.5 x the interquartile range for O_3 at that month. Open circles represent O_3 values that are greater than upper whisker O_3 values. Abbreviations: ppb, parts per billion.

© 2018 Howe CG et al. *JAMA Network Open.*

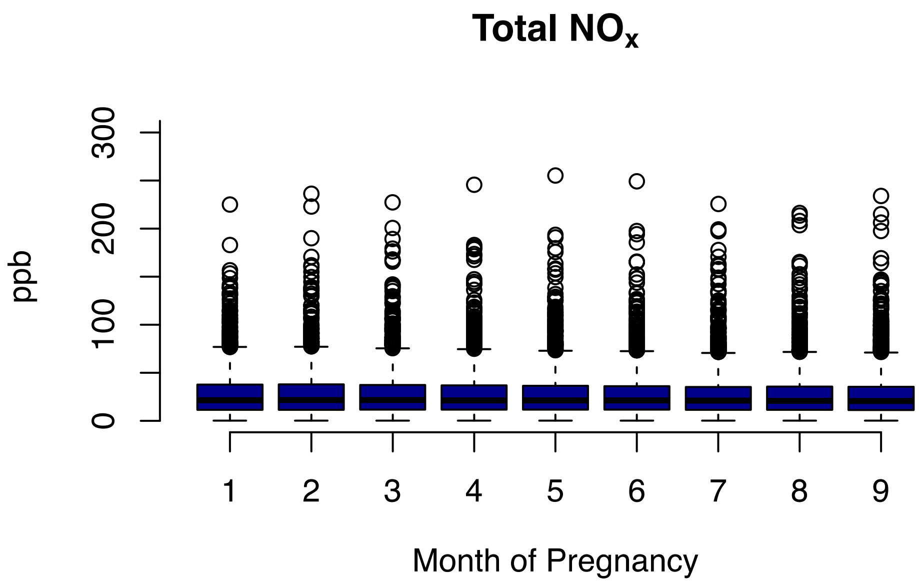

eFigure 6. Box and Whisker Plots of Total Nitrogen Oxides (NO_x) Across Pregnancy. Box and whisker plots showing the distribution of total NO_x (parts per billion (ppb)) at each of the first nine months of pregnancy. The boxes represent the interquartile ranges for Total NO_x. The horizontal black line dividing the box represents the median. Lower whiskers represent the first quartile Total NO_x value - 1.5 x the interquartile range for Total NO_x at that month. Upper whiskers represent the third quartile Total NO_x value + 1.5 x the interquartile range for Total NO_x at that month. Open circles represent Total NO_x values that are greater than upper whisker Total NO_x values. Abbreviations: ppb, parts per billion.

© 2018 Howe CG et al. *JAMA Network Open.*

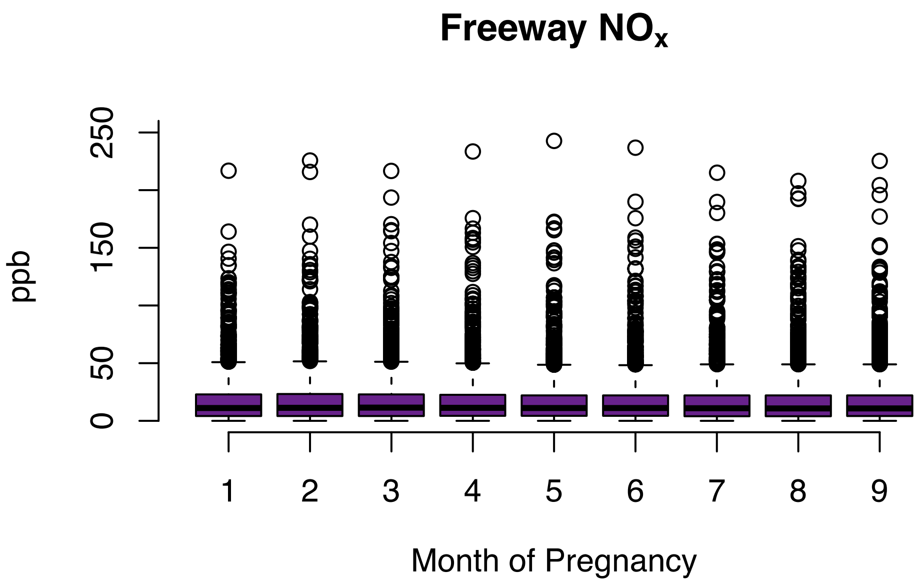

eFigure 7. Box and Whisker Plots of Freeway Nitrogen Oxides (NO_x) Across Pregnancy. Box and whisker plots showing the distribution of freeway NO_x (parts per billion (ppb)) at each of the first nine months of pregnancy. The boxes represent the interquartile ranges for freeway NO_x. The horizontal black line dividing the box represents the median. Lower whiskers represent the first quartile freeway NO_x value - 1.5 x the interquartile range for freeway NO_x at that month. Upper whiskers represent the third quartile freeway NO_x value + 1.5 x the interquartile range for freeway NO_x at that month. Open circles represent freeway NO_x values that are greater than upper whisker freeway NO_x values. Abbreviations: ppb, parts per billion.

© 2018 Howe CG et al. *JAMA Network Open.*

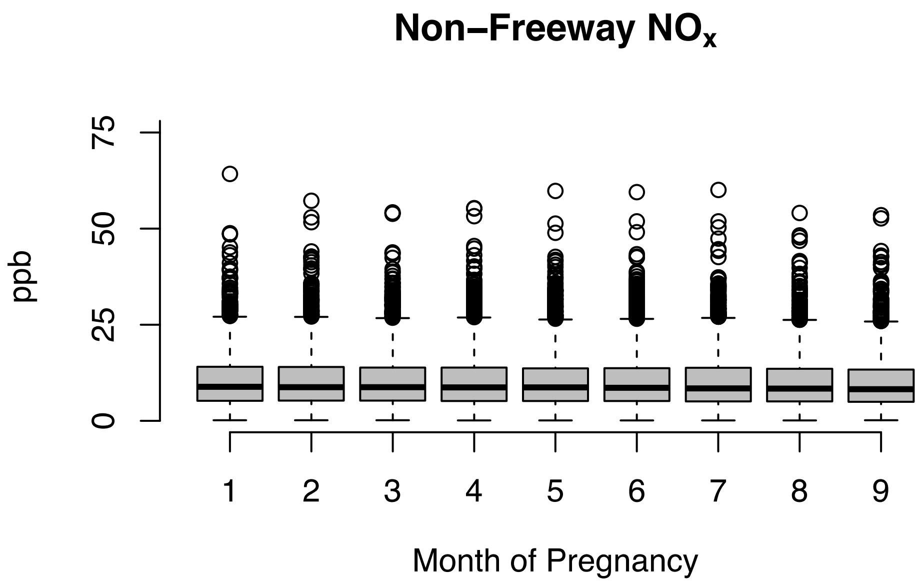

eFigure 8. Box and Whisker Plots of Non-Freeway Nitrogen Oxides (NO_x) Across Pregnancy. Box and whisker plots showing the distribution of non-freeway NO_x (parts per billion (ppb)) at each of the first nine months of pregnancy. The boxes represent the interquartile ranges for non-freeway NO_x. The horizontal black line dividing the box represents the median. Lower whiskers represent the first quartile no-freeway NO_x value - 1.5 x the interquartile range for non-freeway NO_x at that month. Upper whiskers represent the third quartile non-freeway NO_x value + 1.5 x the interquartile range for non-freeway NO_x at that month. Open circles represent non-freeway NO_x values that are greater than upper whisker non-freeway NO_x values. Abbreviations: ppb, parts per billion.

© 2018 Howe CG et al. *JAMA Network Open.*

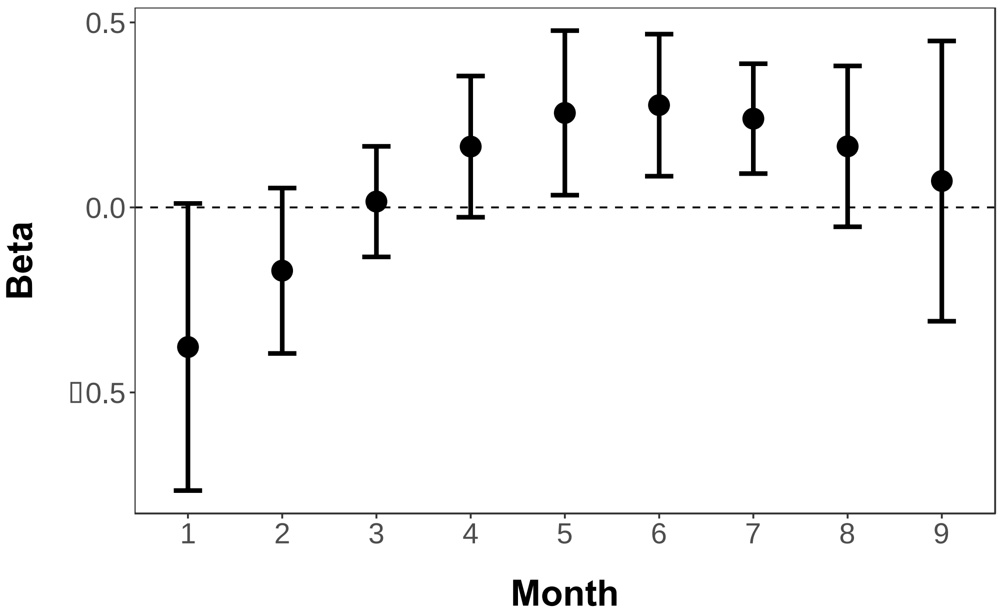

eFigure 9. Distributed Lag Model Results for Nitrogen Dioxide (NO_2). Beta coefficients and 95% confidence intervals from distributed lag models for associations between NO_2 and total thyroxine (TT4) concentrations at birth, adjusted for baby's sex, baby's race/ethnicity, gestational age at birth, season of birth, maternal parity, maternal age, maternal education, maternal tobacco smoke use during pregnancy, age at newborn blood spot collection, and the community of the participant at enrollment, among full-/late-term newborns. A natural cubic spline function with three degrees of freedom was used. Beta coefficients represent the difference in newborn TT4 (μg/dL) for a 2 SD difference in NO_2. N = 1880.

© 2018 Howe CG et al. *JAMA Network Open.*

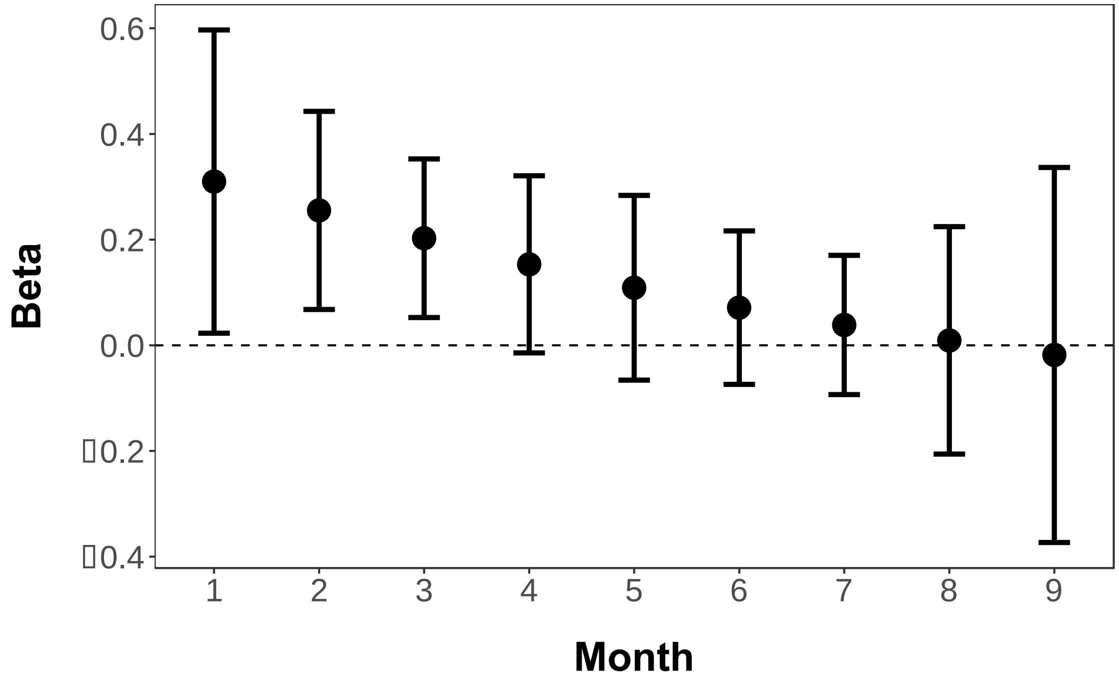

eFigure 10. Distributed Lag Model Results for Ozone (O_3). Beta coefficients and 95% confidence intervals from distributed lag models for associations between O_3 and total thyroxine (TT4) concentrations at birth, adjusted for baby's sex, baby's race/ethnicity, gestational age at birth, season of birth, maternal parity, maternal age, maternal education, maternal tobacco smoke use during pregnancy, age at newborn blood spot collection, and the community of the participant at enrollment, among full-/late-term newborns. A natural cubic spline function with three degrees of freedom was used. Beta coefficients represent the difference in newborn TT4 (μg/dL) for a 2 SD difference in O_3. N = 1880.

© 2018 Howe CG et al. *JAMA Network Open.*

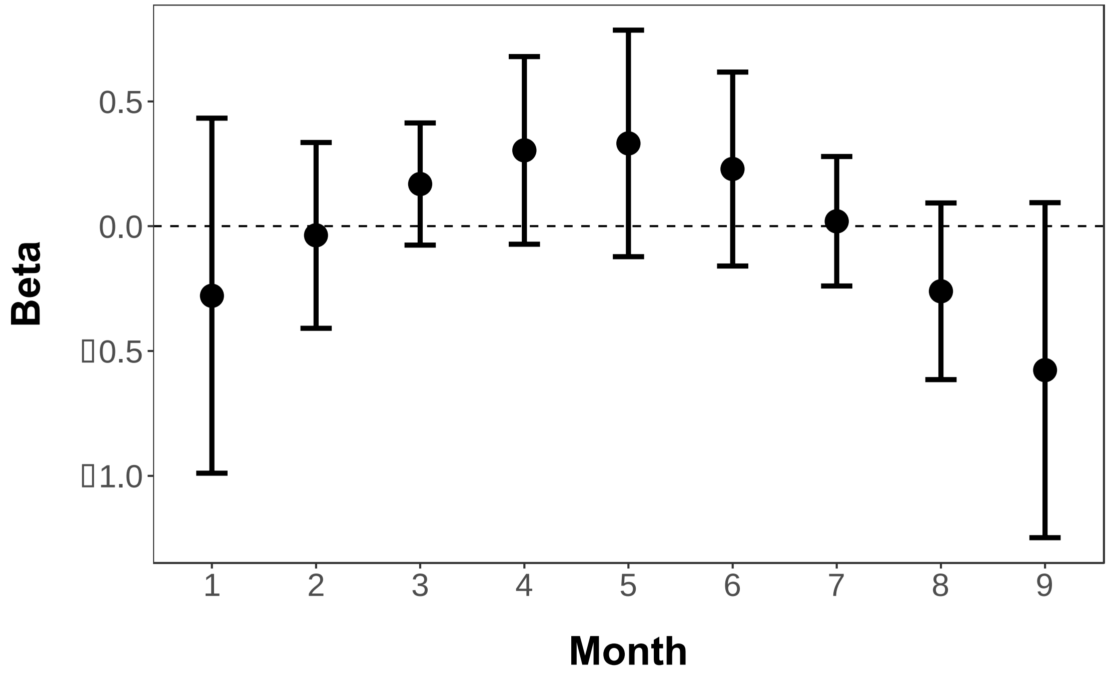

eFigure 11. Distributed Lag Model Results for Total Nitrogen Oxides (NO_x). Beta coefficients and 95% confidence intervals from distributed lag models for associations between total NO_x and total thyroxine (TT4) concentrations at birth, adjusted for baby's sex, baby's race/ethnicity, gestational age at birth, season of birth, maternal parity, maternal age, maternal education, maternal tobacco smoke use during pregnancy, age at newborn blood spot collection, and the community of the participant at enrollment, among full-/late-term newborns. A natural cubic spline function with three degrees of freedom was used. Beta coefficients represent the difference in newborn TT4 (µg/dL) for a 2 SD difference in total NO_x. N = 1824.

© 2018 Howe CG et al. *JAMA Network Open.*

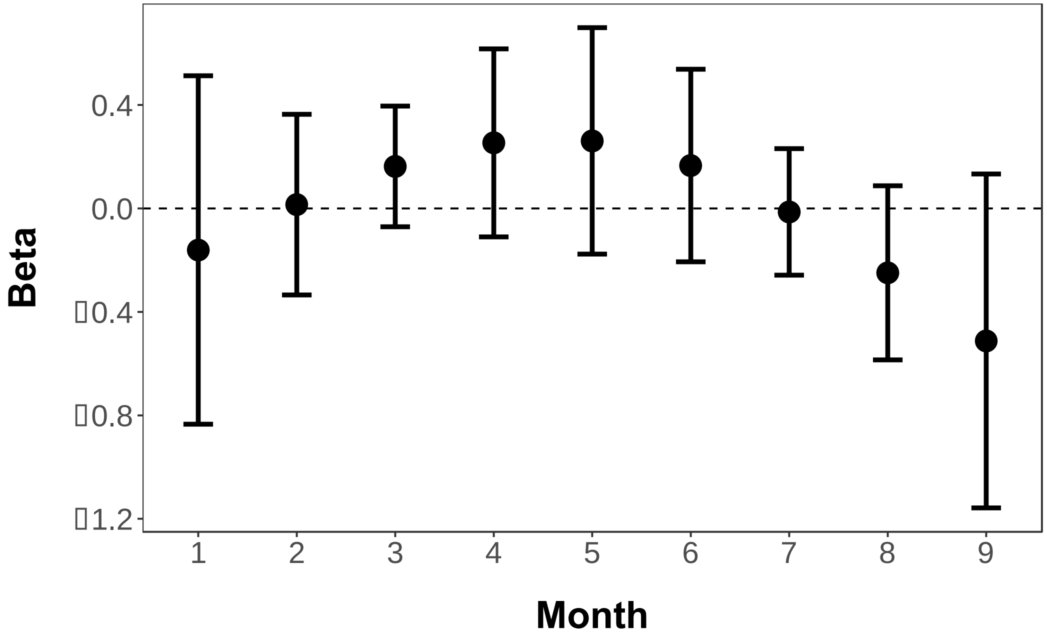

eFigure 12. Distributed Lag Model Results for Freeway Nitrogen Oxides (NO_x). Beta coefficients and 95% confidence intervals from distributed lag models for associations between freeway NO_x and total thyroxine (TT4) concentrations at birth, adjusted for baby's sex, baby's race/ethnicity, gestational age at birth, season of birth, maternal parity, maternal age, maternal education, maternal tobacco smoke use during pregnancy, age at newborn blood spot collection, and the community of the participant at enrollment, among full-/late-term newborns. A natural cubic spline function with three degrees of freedom was used. Beta coefficients represent the difference in newborn TT4 (μg/dL) for a 2 SD difference in freeway NO_x. N = 1824.

© 2018 Howe CG et al. *JAMA Network Open.*

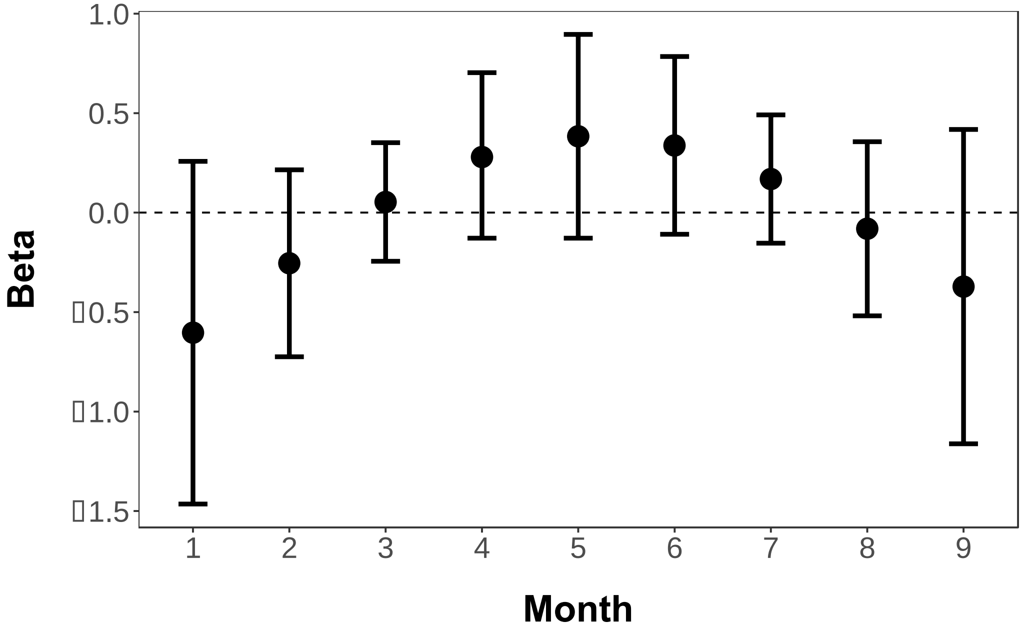

eFigure 13. Distributed Lag Model Results for Non-Freeway Nitrogen Oxides (NO_x). Beta coefficients and 95% confidence intervals from distributed lag models for associations between non-freeway NO_x and total thyroxine (TT4) concentrations at birth, adjusted for baby's sex, baby's race/ethnicity, gestational age at birth, season of birth, maternal parity, maternal age, maternal education, maternal tobacco smoke use during pregnancy, age at newborn blood spot collection, and the community of the participant at enrollment, among full-/late-term newborns. A natural cubic spline function with three degrees of freedom was used. Beta coefficients represent the difference in newborn TT4 (μg/dL) for a 2 SD difference in non-freeway NO_x. N = 1824.

© 2018 Howe CG et al. *JAMA Network Open.*

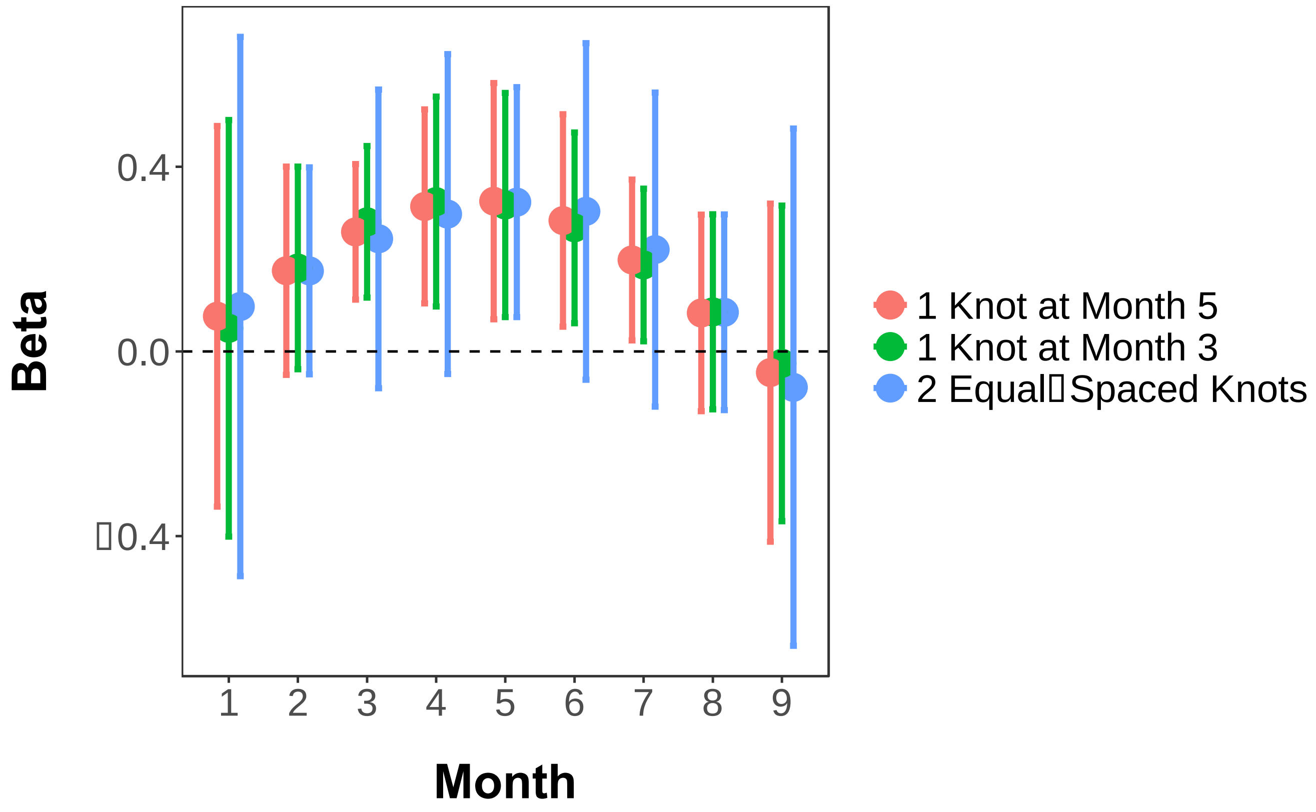

eFigure 14. Sensitivity Analyses for Particulate Matter < 2.5 µm ($PM_{2.5}$) Distributed Lag Models. Comparison of $PM_{2.5}$ distributed lag models results using natural cubic splines with 1 knot, placed at the median lag period, month 5 of pregnancy (pink) versus 1 knot, placed at month 3 of pregnancy (green), versus 2 equal-spaced knots: one between months three and four of pregnancy and another between months six and seven of pregnancy (blue). Estimates and corresponding 95% confidence intervals represent the difference in newborn total thyroxine (µg/dL) for a 2 standard deviation difference in $PM_{2.5}$. All models were adjusted for baby's sex, baby's race/ethnicity, gestational age at birth, season of birth, maternal parity, maternal age, maternal education, maternal tobacco smoke use during pregnancy, age at newborn blood spot collection, and the community of the participant at recruitment, among full-/late-term newborns. n = 1876.

© 2018 Howe CG et al. *JAMA Network Open.*

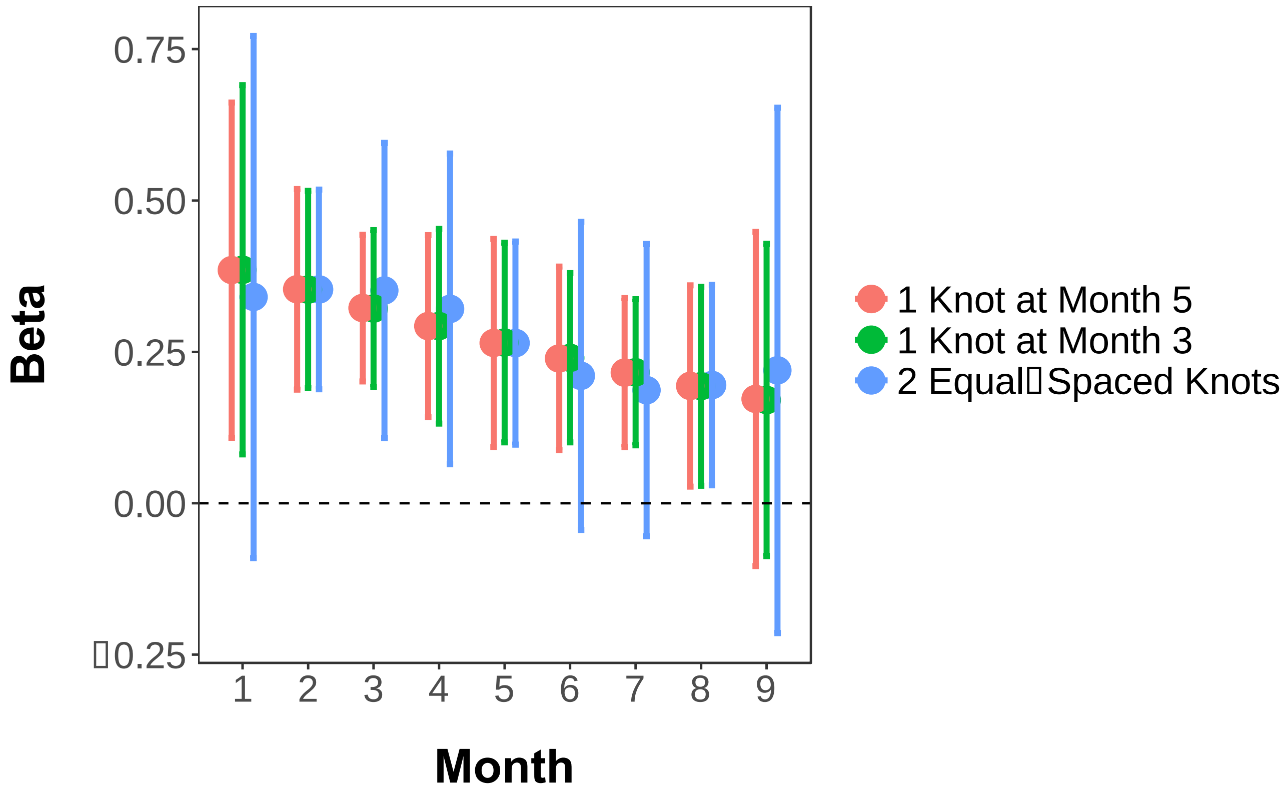

eFigure 15. Sensitivity Analyses for Particulate Matter < 10 µm (PM_{10}) Distributed Lag Models. Comparison of PM_{10} distributed lag models results using natural cubic splines with 1 knot, placed at the median lag period, month 5 of pregnancy (pink) versus 1 knot, placed at month 3 of pregnancy (green), versus 2 equal-spaced knots: one between months three and four of pregnancy and another between months six and seven of pregnancy (blue). Estimates and corresponding 95% confidence intervals represent the difference in newborn total thyroxine (µg/dL) for a 2 standard deviation difference in PM_{10}. All models were adjusted for baby's sex, baby's race/ethnicity, gestational age at birth, season of birth, maternal parity, maternal age, maternal education, maternal tobacco smoke use during pregnancy, age at newborn blood spot collection, and the community of the participant at recruitment, among full-/late-term newborns. n = 1880.

© 2018 Howe CG et al. *JAMA Network Open.*
